# Glycoproteomics Analysis of Triple Wild-Type Lung Adenocarcinoma Tissue Samples

**DOI:** 10.1021/acs.jproteome.4c01063

**Published:** 2025-04-02

**Authors:** Simon Nándor Sugár, Balázs András Molnár, Fanni Bugyi, Gábor Kecskeméti, Zoltán Szabó, Ibolya Laczó, Tünde Harkó, Judit Moldvay, Lilla Turiák

**Affiliations:** † MTA-HUN-REN TTK Lendület (Momentum) Glycan Biomarker Research Group, HUN-REN Research Centre for Natural Sciences, Magyar Tudósok Körútja 2, Budapest H-1117, Hungary; ‡ Hevesy György PhD School of Chemistry, ELTE Eötvös Loránd University, Pázmány Péter Sétány 1/A, Budapest H-1117, Hungary; § Department of Medical Chemistry, Albert Szent-Györgyi Medical School, University of Szeged, Dóm Square 8, Szeged H-6720, Hungary; ∥ Békés County Central Hospital, Semmelweis Utca 1, Gyula, H-5700, Hungary; ⊥ National Korányi Institute of Pulmonology, Korányi Frigyes Street 1, Budapest, H-1121, Hungary; # Pulmonology Clinic, Albert Szent-Györgyi Medical School, University of Szeged, Alkotmány Street 36, Deszk H-6771, Hungary

**Keywords:** Lung adenocarcinoma, FFPE tissue, *N*-Glycoproteomics, Mass spectrometry, Cancer research

## Abstract

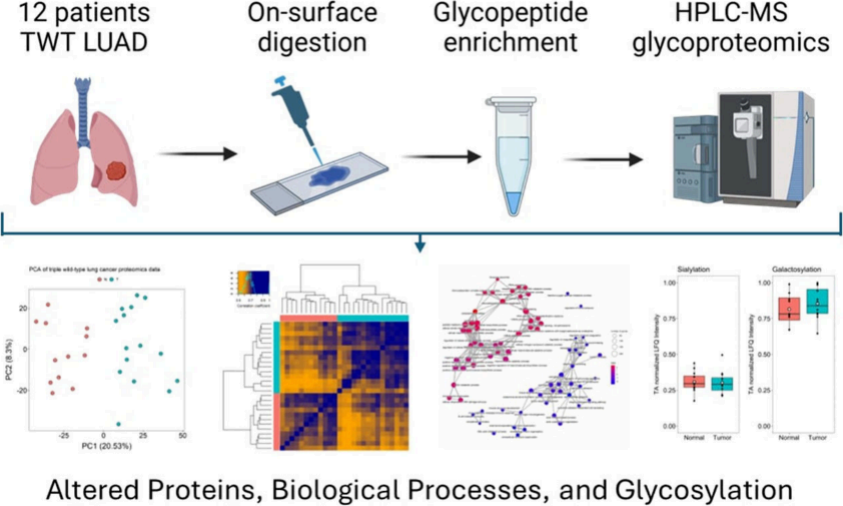

Lung cancer has both high incidence and mortality, making it the leading cause of cancer-related mortality worldwide. It is a highly heterogeneous disease, with several histological subtypes and genetic alterations that influence prognosis and available treatment options. Here, we focus on the triple wild-type (TWT) subtype of lung adenocarcinoma (LUAD) that lacks the three most common actionable genetic alterations, subsequently making targeted therapies inaccessible. In this study, our aim was the mass spectrometry-based proteomic and *N*-glycoproteomic characterization of tumor and adjacent normal lung tissue regions from individuals (*n* = 12) with TWT LUAD. We found several proteins previously identified as potential prognostic or diagnostic biomarkers in LUAD and described dysregulated biological processes, giving an overview of the general differences between healthy and tumor tissue. Also, we highlight specific signatures detected using *N*-glycoproteomics and discuss their potential and importance based on data from databases and literature. To the best of our knowledge, this is the first *N*-glycoproteomics-focused study on TWT LUAD, and it could provide a valuable resource for further studies into this less well characterized subtype of lung cancer. For instance, we report altered *N*-glycosylation for several glycoproteins implicated in LUAD and other cancers that could have functional importance connected to the disease.

## 1 Introduction

Lung cancer is the leading cause of cancer-related mortality worldwide.^1,2^ Its incidence is strongly correlated to smoking, although nonsmokers are also at risk due to factors such as second-hand smoke, air pollution, and genetic predisposition.^3^ Mortality rates for lung cancer are high, accounting for about 1.8 million deaths (18%) worldwide in 2020.^2^ This disease is often diagnosed at an advanced stage, when treatment effectiveness is limited.^4^ The economic impact of lung cancer is substantial, imposing a burden on governments, patients, families, and ultimately the entire society.^5^

Lung cancer is categorized into two main types based on its histology: small cell lung cancer (SCLC) and non-small cell lung cancer (NSCLC). NSCLC makes up about 85% of all lung cancer cases and has three main subtypes: adenocarcinoma (AC), squamous cell carcinoma (SqCC), and large cell carcinoma (LCC). AC is the most common subtype, with 50% of all NSCLC cases, especially among nonsmokers. It often develops in the peripheral lung areas and tends to grow and spread slower than other lung cancers, especially SCLC.^6^ The histological classification of lung cancer is crucial in determining appropriate treatment approaches.^7,8^

Lung adenocarcinoma displays a diverse landscape of genetic alterations that play critical roles in pathogenesis and subsequently disease prognosis and treatment options.^9^ Triple wild-type (TWT) lung adenocarcinoma (LUAD) refers to a subtype that is characterized by the absence of alterations in three key genes: *EGFR* (epidermal growth factor receptor), *ALK* (anaplastic lymphoma kinase), and *KRAS* (Kirsten rat sarcoma viral oncogene).^10^ These genes are frequently mutated or translocated in LUAD and contribute to the growth and spread of cancer cells and are actionable with targeted therapies.^9,11^

The treatment of patients with TWT LUAD can be challenging, as they cannot benefit from targeted therapies that inhibit the effect of these genetic alterations (e.g., gefitinib for *EGFR* mutation,^12^ crizotinib for *ALK* translocation,^13^ and sotorasib for *KRAS* mutation^14^). Instead, treatment for TWT LUAD may rely on other options including chemotherapy, immunotherapy, or a combination of these, depending on several factors.^10,15,16^

Protein structure and subsequent function are heavily influenced by post-translational modifications (PTMs), such as glycosylation, phosphorylation, acetylation, hydroxylation, and ubiquitination. There are two main types of protein glycosylation, *N*- and *O*-glycosylation. During *N*-glycosylation, a carbohydrate residue (glycan) can be attached to asparagine residues (glycosylation sites) corresponding to the consensus sequence (Asn-X-Ser/Thr, where X≠Pro is the amino acid) of proteins. All eukaryotic *N*-glycans have a common core structure, and can be categorized into complex, hybrid, and oligomannose classes.^17^*N*-glycosylation plays an important role in diverse biological processes (e.g., cell–cell interactions),^18^ and aberrant glycosylation is frequently observed in pathophysiological conditions such as lung cancer, where several studies reported changes in *N*-glycosylation from both tissue, serum, and plasma samples.^19^

Understanding the molecular profile of the different types of LUAD, including TWT, is an important step in developing personalized cancer therapy, allowing for treatments that are tailored to the molecular characteristics of an individual’s cancer. In this study, we performed the proteomic and *N*-glycoproteomic characterization of formalin-fixed paraffin-embedded (FFPE) tissue samples from 12 individuals with TWT LUAD and compared the tumor and tumor adjacent normal regions using a previously developed on-surface tryptic digestion protocol.^20^ To the best of our knowledge, this is the first *N*-glycoproteomics-focused study into this rarely studied subtype of lung cancer.

## 2 Methods

### 2.1 Sample Information

FFPE tissue sections were obtained from the National Korányi Institute of Pulmonology. Exclusion criteria included the presence of mutations in *EGFR* and *KRAS* genes as well as rearrangement of the *ALK* gene. Inclusion criteria were lung adenocarcinoma patients TWT for the above-mentioned genetic alterations, age of patients between 45 and 75 and the cohort was balanced for gender (*n* = 6 male, *n* = 6 female), age, and cancer grade (*n* = 6 grade 2, *n* = 6 grade 3). The work was approved by the Medical Research Council (TUKEB), and the number of the ethical permit is IV/2567-4/2020/EKU, 22/04/2020. Patient and sample information is summarized in Table 1.

**Table 1:** Summary of Patient and Sample Information

Sample Characteristics	No. of Patients
Total No.	12
Age	61 (47–74)
Gender	
Male	6
Female	6
Smoking	
Never	2
Former	3
Current	7
Grade	
Grade 2	6
Grade 2–3	1
Grade 3	5

### 2.2 FFPE Tissue Preparation for Analysis

Tissue sections (10 μm thick) were baked at 60 °C for 2 h to prevent tissue detachment. Next, deparaffinization was carried out by sequentially incubating the slides in xylene for 2 × 3 min, in ethanol for 2 × 5 min, in 90:10 (v/v, %) ethanol:water for 3 min, in 70:30 (v/v, %) ethanol:water for 3 min, in 10 mM NH_4_HCO_3_ (water) for 5 min, and finally in water for 1 min. After dewaxing, heat-induced antigen retrieval was performed (95 mM trisodium citrate + 21 mM citric acid in water, pH = 6) for 30 min at 80–85 °C to disrupt cross-linking induced by formalin fixation.

### 2.3 On-Surface Digestion

Digestion was carried out on specific tissue regions of unstained sections based on characterization by a pathologist on parallel tissues slides which were stained with H&E (see Supporting Information Figure S1 for images of the tissue regions analyzed). Selected tissue regions were circumvented using a razor to minimize dispersion of the droplet pipetted on the surface. The proteins were reduced using 0.1% RapiGest and 5 mM dithiothreitol in 3 μL of 20% glycerol for 20 min at 55 °C and then alkylated using 10 mM iodoacetamide in 3 μL of 25 mM ammonium bicarbonate (ABC) buffer and 20% glycerol for 20 min at room temperature in the dark. The digestion was performed cyclically, each one lasting for 40 min at 37 °C in a humidified box with five cycles in total. In the first two cycles, endoproteinase LysC-trypsin mixture was added in ca. 1:25 ratio, in 3 μL of 50 mM ABC and 20% glycerol. Subsequently, in the last three cycles, trypsin was added in a 1:5 ratio, in 3 μL of 50 mM ABC, and 20% glycerol. After the digestion steps, the extraction of the protein digest was carried out by pipetting 3 μL of 10% acetic acid extraction solvent five times on the digested spots. Peptide extracts were then dried down and stored at–20 °C until further usage.

### 2.4 Glycopeptide Enrichment

Glycopeptide enrichment was performed using acetone precipitation.^21^ The solubility of nonglycosylated and glycosylated peptides are different in acetone, resulting in a pellet fraction rich in glycosylated peptides and most peptides remaining in solution. In a previous study we have optimized the acetone precipitation protocol for small sample amounts resulting in an approximately 10-fold enrichment of *N*-glycopeptides.^22^ Samples were reconstituted in 15 μL of 1% FA, 150 μL of ice-cold acetone was added, and samples were stored at −20 °C overnight. Samples were then centrifuged at 13000*g* for 10 min, and then the supernatants were removed, dried down, and stored at −20 °C for subsequent proteomics analysis. The pellet fractions were dried down briefly (5 min), then resuspended in 10 μL of injection solvent and subsequently stored in the autosampler unit for *N*-glycoproteomics analysis.

### 2.5 Solid Phase Extraction Cleanup

C18 spin columns (Thermo Fisher Scientific, Waltham, MA, USA) were used for desalting and cleanup of the supernatant fractions. After the column was conditioned, washed, and equilibrated, the sample was loaded onto the column in 0.1% heptafluorobutyric acid (HFBA) in water. The elution was performed with 30:70 (v/v, %) water:ACN. After the elution, the samples were dried down and stored at −20 °C until further usage.

### 2.6 HPLC-MS Analysis

A Waters ACQUITY UPLC M-Class (Milford, MA, USA) system coupled to a Thermo Fisher Exploris 240 Orbitrap mass spectrometer was used for analysis of the samples.

Trapping was performed on a Symmetry C18 (100 Å, 5 μm, 180 μm × 20 mm, Waters, Budapest, Hungary) trap column, while separation of peptides and *N*-glycopeptides was achieved on an Acquity M-Class BEH130 C18 (1.7 μm, 75 μm × 250 mm, Waters, Budapest, Hungary) analytical column. Eluent A was 99.9% H_2_O + 0.1% FA, eluent B was 99.9% ACN + 0.1% FA, with a gradient program of the following: 2–25% B from 2 to 82 min, then 25–40% B from 82 to 85 min, then 40–90% B from 85 to 86 min, then 90–2% B from 88 to 90 min.

For the proteomics and *N*-glycoproteomics measurements, capillary temperature was set at 275 °C, nebulizer, and carrier gas at 0, capillary voltage at 1.8 kV, in positive mode. For proteomics, the resolution was set at 70000, with an AGC of 10^6^, maximum injection time of 120 ms, for the mass range of 360–2200 Da. In MS/MS mode, the isolation window was set at 2 Da, with stepwise HCD fragmentation at 27–30–32 eV, a resolution of 17500, an AGC of 5 × 10^5^, and a maximum injection time of 60 ms, for the mass range of 200–2000. Minimum AGC was set at 10^3^, with a minimum precursor intensity of 1.7 × 10^4^. For glycoproteomics, the resolution was set at 120000, with an AGC of 2 × 10^6^, a maximum injection time of 200 ms, for the mass range of 360–2200 Da. In MS/MS mode, the isolation window was set at 2 Da, with stepwise HCD fragmentation at 10–20–30 eV, a resolution of 120000, an AGC of 2 × 10^5^, and a maximum injection time of 200 ms, for the mass range of 200–2000. Minimum AGC was set at 10^3^, with a minimum precursor intensity of 1.7 × 10^4^.

### 2.7 Software

Byonic 3.8 and Preview,^23^ MaxQuant 1.7,^24^ GlycReSoft 0.4,^25^ R 3.6.1,^26^ RStudio 1.2,^27^ Microsoft 365.^28^ R packages used: tidyverse,^29^ gplots,^30^ MSnbase,^31^ MSnSet.utils (available at https://github.com/vladpetyuk/vp.misc—accessed October 2024), imputeLCMD,^32^ limma,^33^ edgeR,^34^ and clusterProfiler.^35^ Software settings are provided in Table S1.

### 2.8 Data Analysis

For proteomics, protein identification and quantitation were done using MaxQuant on a focused *Homo sapiens* database, made from merging Byonic search results (search parameters were determined using Preview) from all proteomic analyses. The MaxQuant output was first filtered for Reverse and Contaminant protein groups and then for missing values (found in at least 2/3 of either tumor or adjacent samples). Missing values were imputed using the QRILC method from the package imputeLCMD. Subsequently, differential expression analysis was carried out using limma and edgeR (empirical Bayes moderated *t*-statistics test) with a 1% false discovery rate (FDR) using the Benjamini–Hochberg method. Based on the *t*-statistic values obtained, gene set enrichment analysis (GSEA) was performed using clusterProfiler for Gene Ontology Biological Process (GOBP) terms. Terms were filtered based on semantic similarity and visualized using tree and network plots also using clusterProfiler.

For glycoproteomics, Byonic was used to identify *N*-glycopeptides from all analyses (search parameters were determined with Preview) to create a focused FASTA database containing 699 glycoproteins. This was used to create the search space for GlycReSoft combined with the built-in consensus human *N*-glycan database that includes “biosynthetically feasible N-glycans using enzymes commonly found in humans and limited to at most 26 monosaccharides” (448 entries). The GlycReSoft results were filtered using a cutoff of 3.0 for MS1 score, and 5.0 for MS2 score as previously suggested by others.^36^ Results were then combined and filtered, and glycosylation metrics were calculated using custom scripts in R. Differences in *N*-glycopeptide abundances were determined using parametric and nonparametric two-sample statistical tests (Student’s *t*-test, Welch *t*-test, or Wilcoxon test) determined by checking for normality (Shapiro test) and variance equality (Levene test)) between groups. FDR was controlled using the Benjamini–Hochberg method.

### 2.9 Data Availability

All HPLC-MS data are available online at MassIVE database with the data set ID MSV000096450. The scripts used for both proteomics and glycoproteomics data analysis are available on GitHub at https://github.com/rozmarakiraly/TWT_LUAD_paper_data_analysis.

## 3 Results

**Figure 1:**
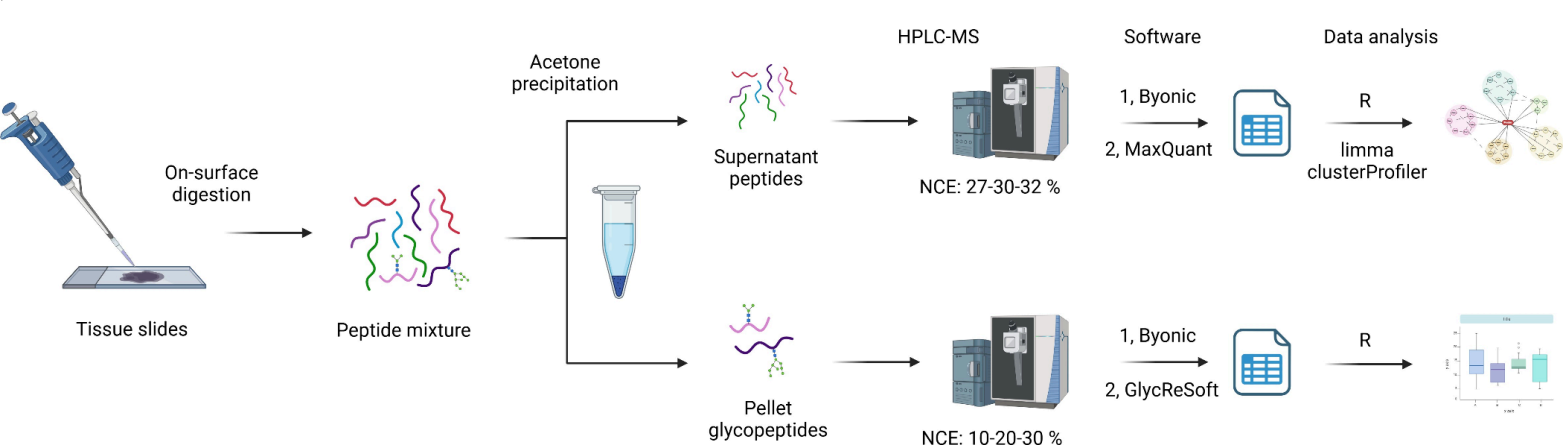
Analysis workflow.

In this study, FFPE tissue sections from patients diagnosed with TWT LUAD were analyzed. Proteomic and *N-*glycoproteomic characterization was performed on small regions from both the tumor- and tumor-adjacent regions of 12 individuals. The sample preparation and measurement workflow are presented in Figure 1.

**Figure 2:**
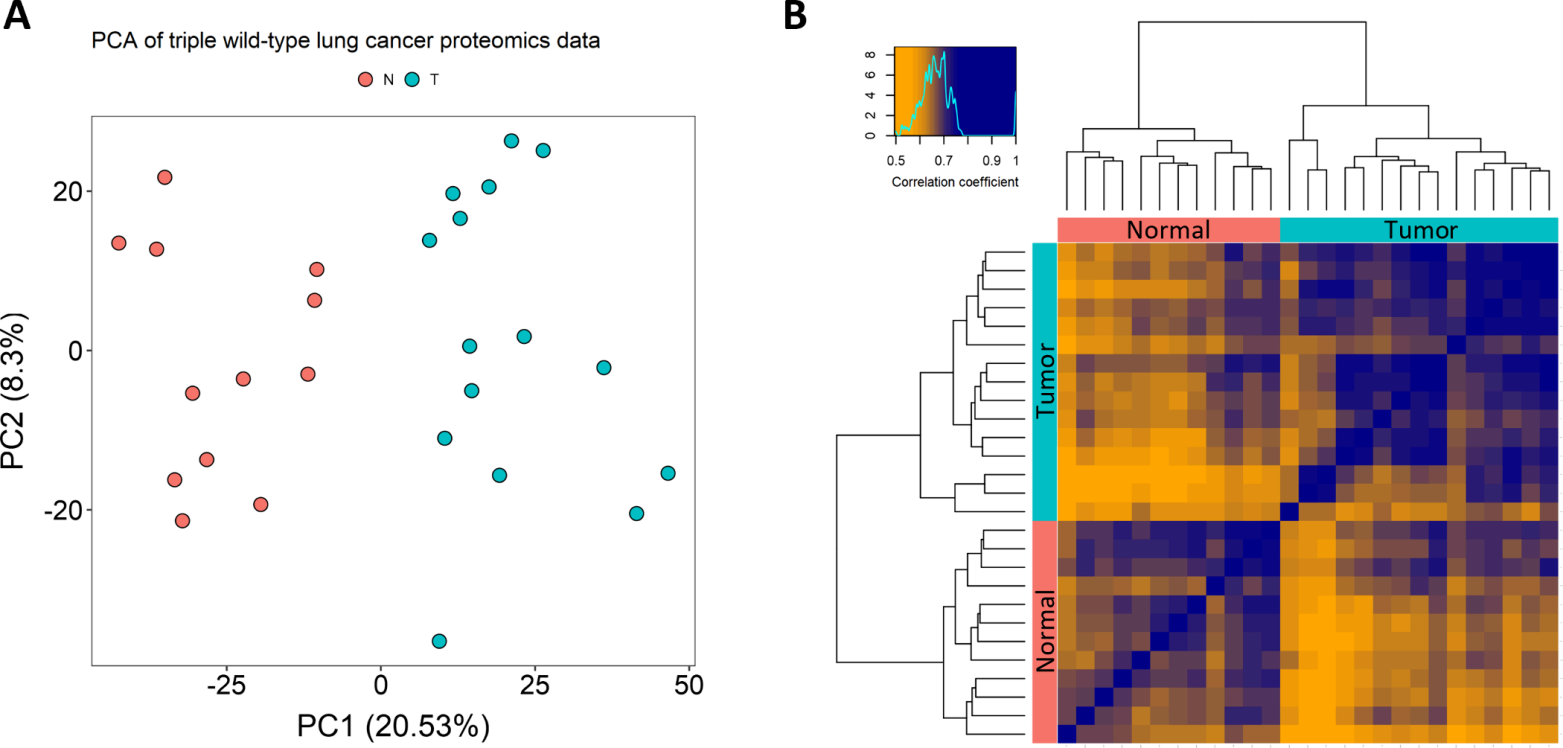
(A) Principal component analysis of tumor adjacent normal (red) and tumor (blue) samples. (B) Sample-wise correlation heatmap of tumor adjacent normal (red) and tumor (blue) samples.

In the proteomics analysis using data dependent acquisition (DDA) we identified 3972 proteins in total and approximately 1800 per sample using Byonic. Using these proteins as a search space, 3376 proteins were quantified using MaxQuant and then 2284 of them were selected through multiple filtering steps for further analysis (for details see Section 2.8). Initial assessment of the data showed no batch effects, and principal component analysis and sample-wise correlation—shown in Figure 2—demonstrated that intragroup differences were considerably higher than intergroup differences.

**Figure 3:**
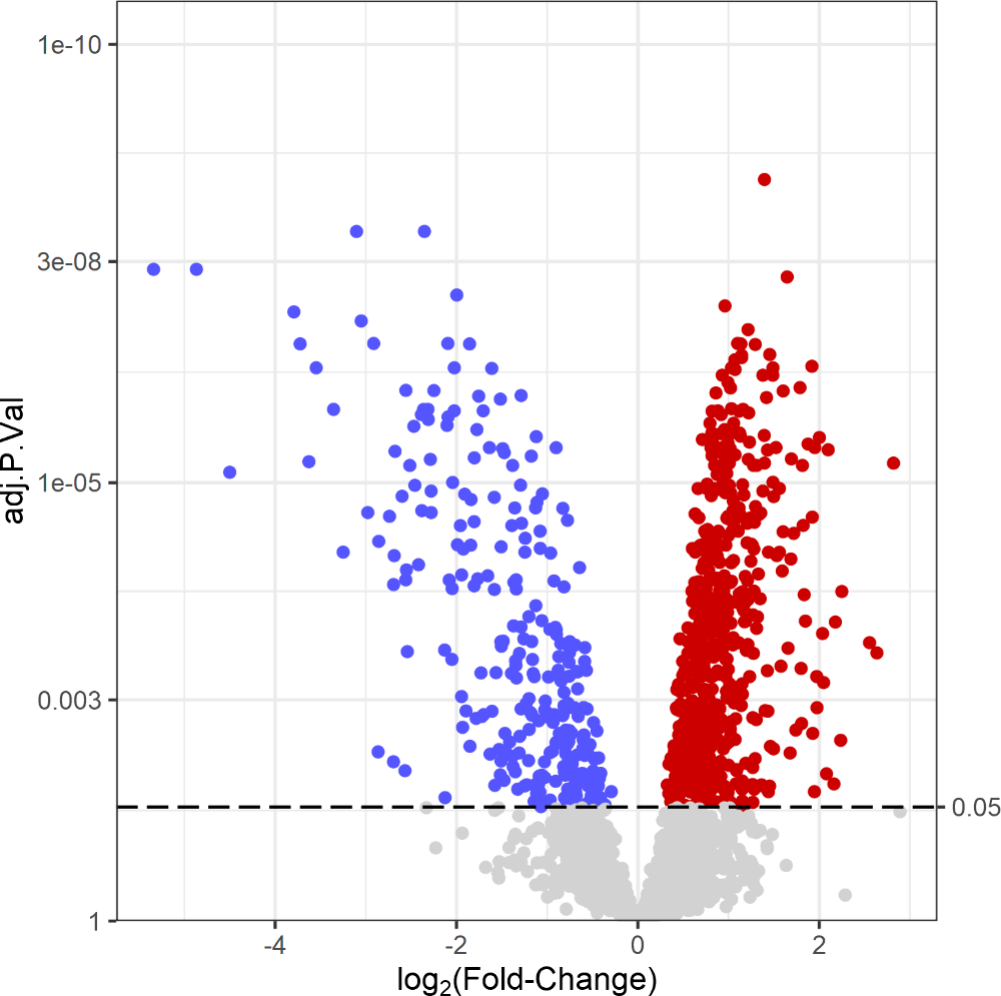
Volcano plot of protein Fold-Change values (*x*-axis, log 2 transformed) vs adjusted *p*-values (*y*-axis). Proteins overexpressed in tumor regions compared to adjacent normal are red; those underexpressed in tumor regions compared to adjacent normal are blue. Proteins with not statistically significant changes (below the 0.05 FDR threshold) are gray.

To reveal proteins with significant changes in abundance between conditions, limma *t* tests were used, with the False Discovery Rate (FDR) controlled at 0.05 using the Benjamini–Hochberg method, which resulted in the identification of 1066 such proteins (for the complete list of proteins, see Supporting Information Table S2). Out of these proteins 838 showed higher, while 228 showed lower expression in the tumor region compared to the adjacent normal region, shown on Figure 3.

Among the proteins with the largest fold change values that had higher expression in the tumor regions based on the statistical analysis were double-strand break repair protein MRE11, DNA replication licensing factor MCM6 and Protein phosphatase 1G. These proteins have all been previously linked to lung cancer. Likewise, proteins with lower expression in the tumor regions that have been associated with lung cancer have also been identified such as Cadherin-13 and Cartilage acidic protein 1. Furthermore, diagnostic or prognostic markers identified in previous studies were also among the significantly altered proteins, including Small ubiquitin-related modifier 1 (SUMO-1) and Periostin.

**Figure 4:**
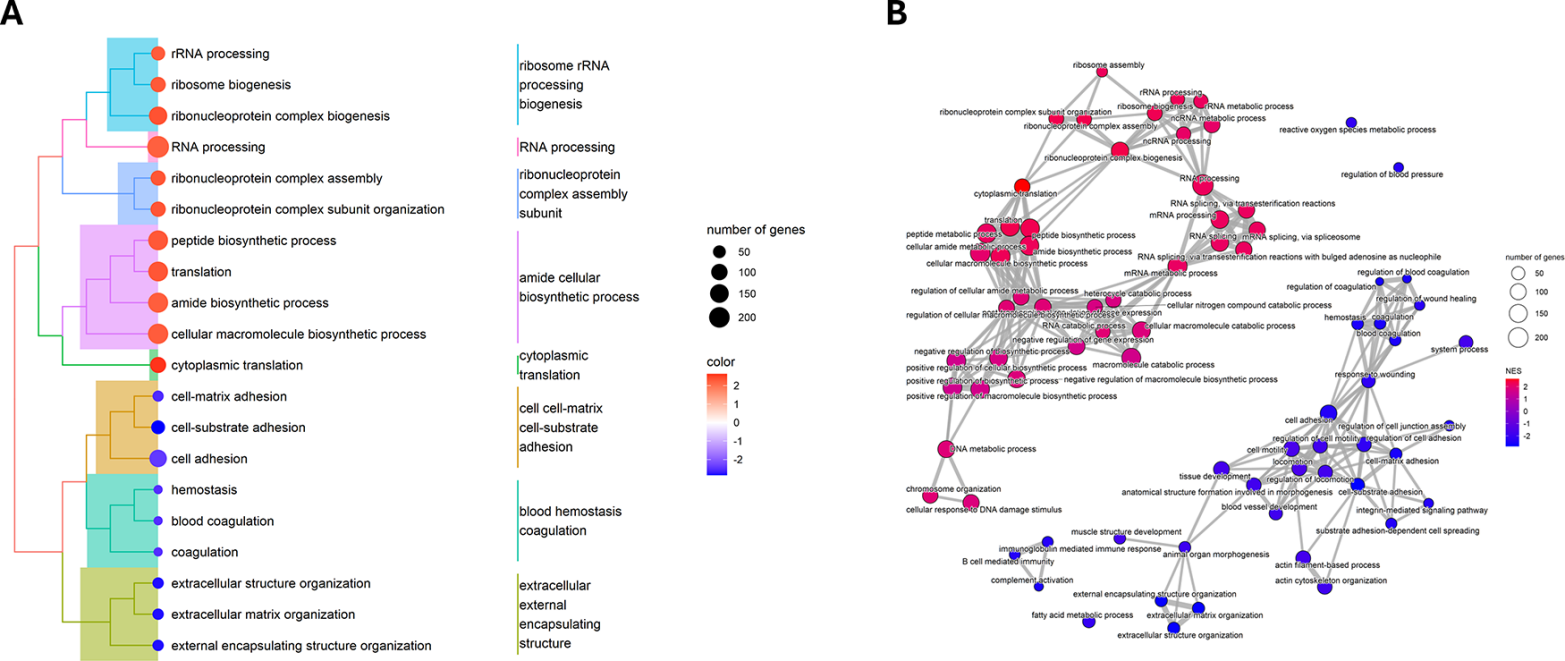
(A) Treeplot of the top 20 enriched GOBP terms based on adjusted *p*-values. Clustering is based on the overlap between gene sets, nodes are colored based on Normalized Enrichment Scores (NESs), and the size of the nodes represents the number of genes in the gene set. The names of the individual clusters are based on the gene sets within. (B) Enrichment map of the top 75 enriched GOBP terms based on adjusted *p*-values. Edges represent the overlap between data sets, nodes are colored based on NESs, and the size of the nodes represents the number of genes in the gene set.

Based on the results from the two-sample test, Gene Set Enrichment Analysis (GSEA) was performed to aggregate protein-level information and reveal differentially regulated biological processes (for the complete table, see Supporting Information Table S3). GSEA was based on the t-statistic values from the limma *t*-test, the top Gene Ontology Biological Processes (GOBP) based on adjusted p-value, are shown on Figure 4.

Positive NES values correspond to gene sets (Gene Ontology Biological Processes) over-represented at the top of the ranked gene list (proteins overexpressed in tumor regions), while negative NES values correspond to gene sets over-represented at the bottom of the ranked gene list (proteins underexpressed in tumor regions). Top GOBP terms with a positive NES include RNA processing, ribonucleoprotein complex assembly, amide cellular biosynthetic process, and cytoplasmic translation. Top GOBP terms with negative NES are related to cell adhesion, blood coagulation, and extracellular matrix organization.

In the *N*-glycoproteomics analysis, a total number of 1626 unique *N*-glycopeptides were identified using GlycReSoft (for the complete merged output, see Supporting Information Table S4), corresponding to 365 *N*-glycosylated proteins. A large proportion of these *N*-glycopeptides were found in only a small fraction of all measured samples (1099 or 68% in a single sample), so stringent filtering was applied to focus on confident *N*-glycopeptide hits (found in at least 5 samples in one of the groups). This narrowed down the *N*-glycopeptides of interest to 65, corresponding to 33 *N*-glycosylated proteins.

**Figure 5:**
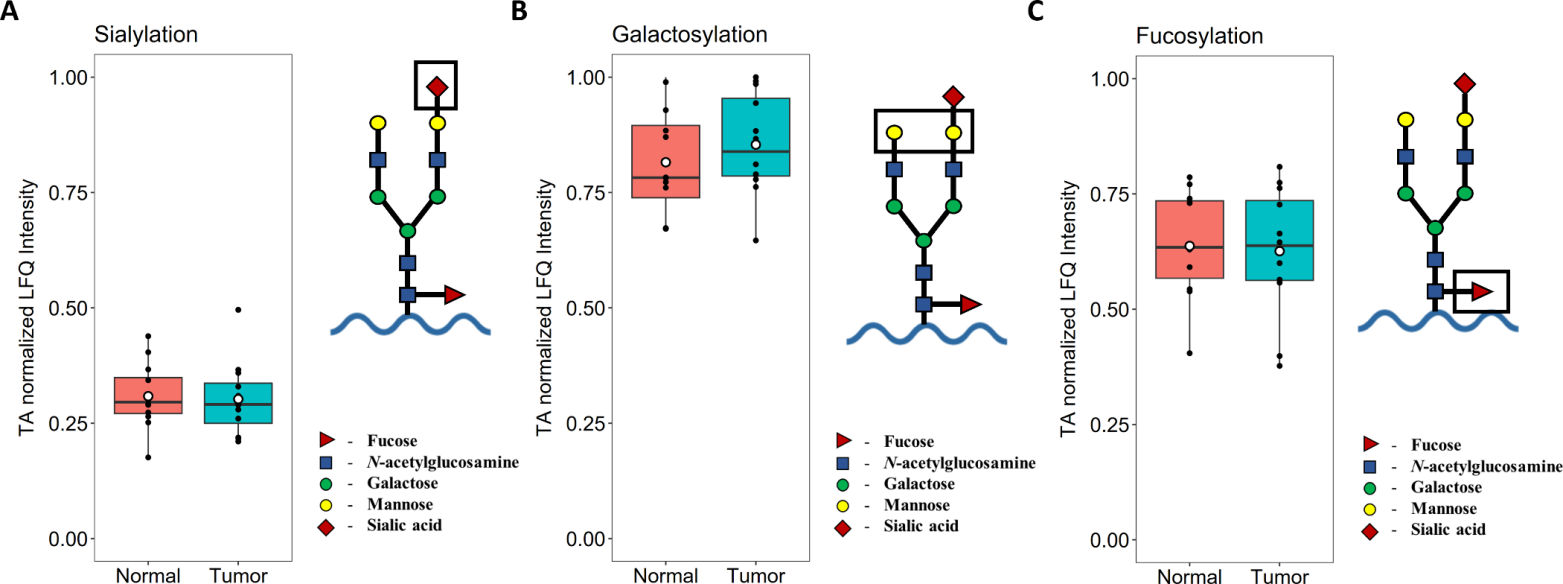
Overall *N*-glycosylation metrics for the tumor and tumor adjacent tissue. Metrics were weighted with *N*-glycopeptide expression and averaged across all of the *N*-glycopeptides. (A) Overall sialylation and an example *N*-glycopeptide with two antennae, one with sialic acid and one without—a sialylation of 0.5. (B) Overall galactosylation and an example *N*-glycopeptide with two antennae, both containing galactose units—a galactosylation of 1.0. (C) Overall fucosylation and an example *N*-glycan with core fucosylation—a fucosylation of 1.0.

In our data set, no significant differences were identified in overall *N*-glycosylation metrics (for more information, see Supporting Information eqs S1–S6) such as sialylation—the proportion of sialylated antennae; galactosylation—the proportion of galactosylated antennae; and fucosylation—the proportion of fucosylated *N*-glycopeptides, shown with examples for the calculation of each metric on Figure 5.

**Figure 6:**
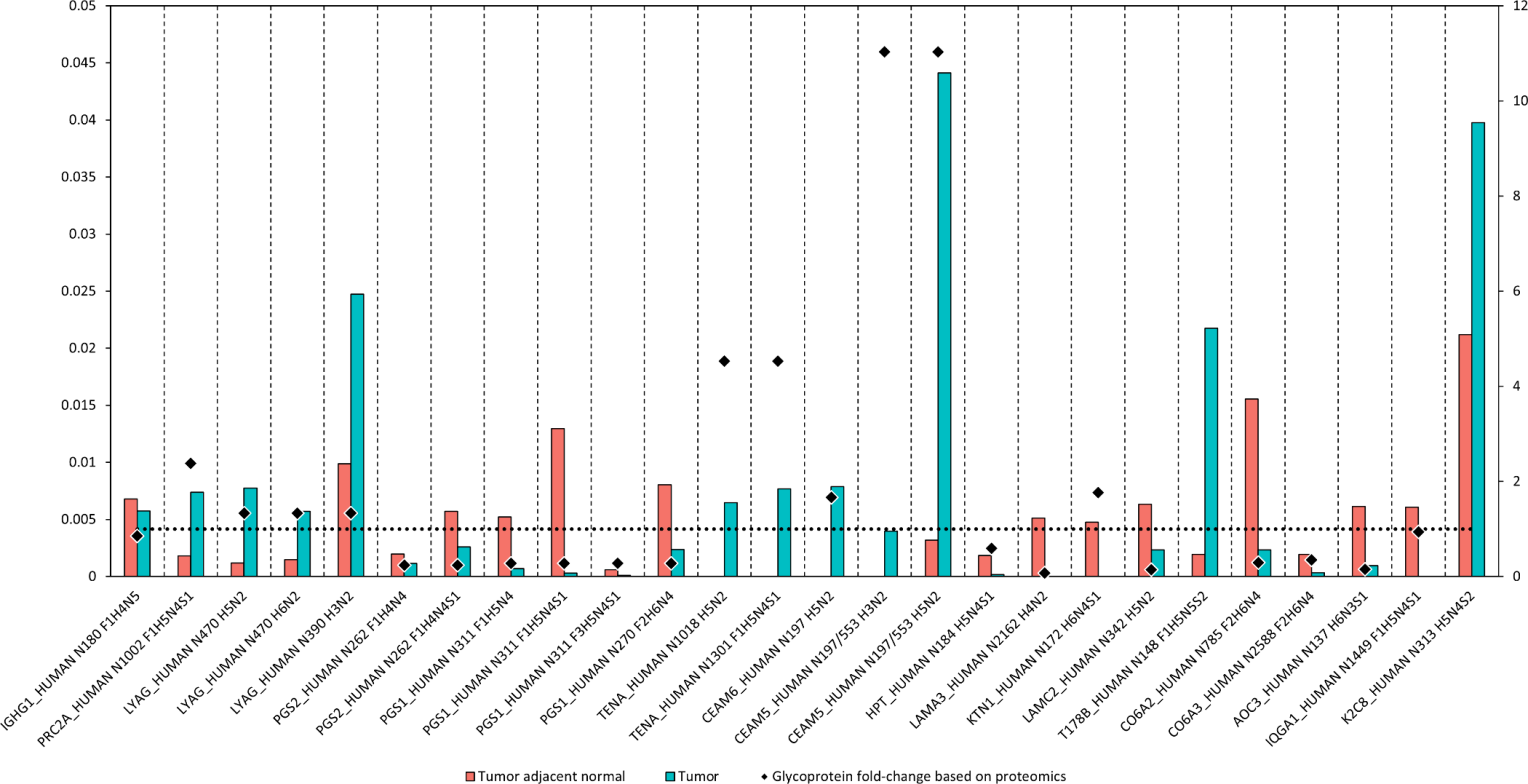
Changes in *N*-glycopeptide abundance between tumor adjacent normal and tumor samples (red and blue bars, respectively), and *N*-glycoprotein fold-change values (black diamonds). The secondary *y*-axis (from 0 to 12) shows protein fold-changes, and the dotted line at 1 shows the threshold for underexpression (fold-change under 1) and overexpression (fold-change over 1) in tumor regions. The nomenclature used for glycopeptides includes the UniProt short name for the protein, the location of the *N*-glycosylated asparagine in the sequence, and the attached glycan. The glycan name includes the number of fucose (F), hexose (H), *N*-acetylhexosamine (N), and sialic acid (S) residues.

Two-samples tests were used to compare the abundance of *N*-glycopeptides between the tumor and adjacent regions, and 26 of them showed statistically significant differences. The fold-changes of the corresponding *N*-glycoproteins (18 in total) were overlaid on *N*-glycopeptide abundances, and the direction (and extent) of changes compared is shown on Figure 6.

Based on Figure 6, the changes in *N*-glycopeptide abundances can be attributed to a combination of changes in *N*-glycosylation and *N*-glycoprotein expression in most cases. For example, the increase of N197/553 H3N2 and H5N2 on CEAM5 in tumor regions can be partially due to the overexpression of CEAM5 itself; on the other hand, the decrease of IQGA1 N1449 F1H5N4S1 is likely affected only by *N*-glycosylation events. The *N*-glycopeptides with increased abundance in tumor regions carried approximately equal parts complex and high-mannose glycans (4 and 5), while *N*-glycopeptides with decreased abundance in tumor regions carried predominantly complex glycans (12 out of 15).

## 4 Discussion

The proteomics analysis of the tumor and tumor adjacent normal regions of TWT LUAD samples revealed several proteins significantly altered that have been shown to be important in the development of lung cancer or identified as potential diagnostic or prognostic markers in previous studies.

Double-strand break repair protein MRE11 is an important DNA damage protein and activator of the Serine-protein kinase ATM protein during the process of double-strand DNA break. Activation of this kinase may have biomarker potential in lung cancer.^37^ Involvement of MRE11 in the IL/6/STAT3 pathway has also been investigated, which is a key pathway in tumor metastasis.^38^ This protein was found to be significantly upregulated in tumor tissue in our study, further highlighting the importance of this protein as an attractive target for lung cancer therapy.

MCM6 is a member of the minichromosome maintenance protein complex (MCM), whose main function is to couple DNA regulation to cell cycle progression and checkpoint regulation.^39^ In a transcriptomic study the prognostic value of different members of the MCM complex in NSCLC patients has been investigated.^40^ Worse overall survival was found in case of higher expression levels of MCM1/2/3/4/5/6/7/8/10.^40^ Our proteomics results revealed significantly higher levels of MCM6 protein in tumor tissue, corroborating transcriptomic data and suggesting therapeutic potential.

Protein phosphatase 1G is encoded by the PPM1G gene, which was previously found to be highly expressed in LUAD compared to normal tissue^41^ in line with our results. It may be an attractive target for LUAD treatment as PPM1G was shown to dephosphorylate MKE6 thereby reducing p38 MAP kinase phosphorylation contributing to the proliferation, invasion and metastasis of LUAD.^41^

SUMO-1 and Periostin have been previously identified as diagnostic and prognostic markers and were among the significantly altered proteins in this study (both overexpressed in tumor samples). SUMO-1 expression levels have been found to be elevated in NSCLC tissues compared to adjacent normal tissue using immunofluorescence.^42^ In a follow-up study it was demonstrated in NSCLC cells that SUMO-1 regulates NF-κB and thereby promotes proliferation and invasion.^43^ Periostin is secreted by fibroblasts and has already been linked to promoting tumor development. It was identified as a negative prognostic factor and potential target in NSCLC increasing tumor proliferation via ERK signaling.^44^

Proteins with lower expression in the tumor regions that have been associated with lung cancer have also been identified in the proteomics analysis such as Cadherin-13 and Cartilage acidic protein 1. Down-regulation of Cadherin-13, a cell adhesion protein in lung cancer cells has been linked with worse prognosis.^45^ Gene expression analysis identified CRTAC1 gene as a potential diagnostic and prognostic biomarker for LUAD.^46^ Cellular experiments also confirmed that LUAD cell proliferation, invasion and migration was reduced in case of CRTAC1 upregulation.^46^ This gene encodes a Cartilage acidic protein a 1, an extracellular matrix protein, which was detected in significantly lower levels in tumor regions in our study.

The results of the proteomics analysis also revealed several differentially regulated biological processes well-known in cancer^47^ such as terms connected to increased proliferation: activated ribosome production and peptide biosynthesis; or the breaking down of the extracellular matrix (ECM): suppressed cell–cell and cell-matrix adhesion, and ECM organization.

The high correlation between our proteomics results and previous studies confirms the validity and robustness of our results and lay the foundation for the *N*-glycoproteomics analysis. In the following, we will discuss the *N*-glycoproteins included in Figure 6 corresponding to the 26 *N*-glycopeptides with different levels in the tumor and adjacent regions. For each *N*-glycoprotein mRNA (from the TCGA data set) and antibody (Ab) staining data from the Pathology Atlas of the Human Protein Atlas (HPA) are provided, and most important changes are discussed.

Based on classification from the HPA, several of the 18 *N*-glycoproteins whose corresponding glycopeptides were significantly altered in the present study are unfavorable cancer markers, and are secreted to either the blood, or the ECM, summarized in Table 2. Based on data from the TCGA data set, four glycoproteins are unfavorable prognostic markers of LUAD. Laminin subunit gamma-2 (LAMC2), with a 5 year survival of 34% for high, and 48% for low LAMC2 expression (*n* = 994 individuals), Haptoglobin (HPT) with a 5 year survival of 30% for high, and 64% for low HPT expression (*n* = 105), Laminin subunit alpha-3 (LAMA3), with a 5 year survival of 28% for high, and a 44% for low LAMA3 expression (*n* = 497 individuals), and Keratin 8 (KRT8) with a 5 year survival of 22% for high, and 49% for low KRT8 expression (*n* = 497).

**Table 2:** Summary of Multiple Classifications for the *N*-Glycoproteins Shown on Figure 6 from the Human Protein Atlas^a^

		RNA					
		Consensus (Human Tissue)		TCGA (Cancer Tissue)			
Protein	Gene	Tissue Specificity	Detected In	Tissue Specificity	Detected In	Prognostic Marker	Blood Atlas: Upregulated in Disease
LYAG	GAA	Low	All	Low	All		
CEAM5	CEACAM5	Enriched (Intestine)	Many	Enchanced (Colon AC, Rectum AC)	Many	UF: LiverC	LungC, ColorectalC, Alcohol-Related Liver Disease
PGS1	BGN	Enhanced (Heart Muscle)	Many	Low	All	UF: ColorectalC, Glioma, Melanoma, RenalC	No
KTN1	KTN1	Low	All	Low	All	UF: BreastC, Head and NeckC, LiverC, RenalC	
LAMC2	LAMC2	Enhanced (Urinary Bladder)	Many	Enchanced (Head and Neck SqCC)	Many	UF: LungC (AD, SqCC), PancreaticC	
AOC3	AOC3	Enhanced (Adipose Tissue)	All	Low	All	F: PancreaticC; UF: LungC (SqCC), RenalC	Several
CEAM6	CEACAM6	Enhanced (Bone Marrow, Esophagus, Intestine, Lung, Salivary Gland)	Many	Enchanced (Lung AC, Rectum AC)	Many		
T178B	TMEM178B	Enhanced (Brain, Heart Muscle, Parathyroid Gland, Tongue)	Many	Enchanced (Glioblastoma Multiforme, Kidney Renal PCC)	Many	F: PancreaticC	
CO6A3	COL6A3	Enhanced (Smooth Muscle)	Many	Low	All	UF: RenalC	Several
IQGA1	IQGAP1	Low	All	Low	All	F: BreastC; UF: PancreaticC, RenalC	-
PGS2	DCN	Enhanced (Ovary)	All	Low	All	UF: RenalC, StomachC	No
HPT	HP	Enriched (Liver)	Many	Enriched (Liver Hepatocellular Carcinoma)	Many	F: LiverC; UF: LungC (AC), RenalC, StomachC	
CO6A2	COL6A2	Low	Many	Low	All	UF: Glioma, RenalC (KIRC, KIRP)	
TENA	TNC	Enhanced (Lymphoid Tissue, Smooth Muscle)	All	Low	All	UF: OvarianC	Several
LAMA3	LAMA3	Low	Many	Enchanced (Head and Neck SqCC)	Many	UF: LungC (AC), PancreaticC	
K2C8	KRT8	Enhanced (Intestine, Stomach)	Many	Low	All	UF: LungC (AC), PancreaticC	
IGHG1	IGHG1	Group Enriched (Lymphoid Tissue, Urinary Bladder)	Many	Low	All	F: CervicalC, Head and neckC; UF: RenalC	
PRC2A	PRRC2A	Low	Many	Low	Many	UF: LiverC	

a RNA-based data include tissue specificity, and tissues detected in from the Consensus dataset, and tissue specificity, tissues detected in and prognostic marker status for the The Cancer Genome Atlas (TCGA) dataset. Finally, information from the Blood Atlas is also included: upregulated in disease. The abbreviations used are the following: UF, unfavorable, F, favorable; C, cancer; AC, adenocarcinoma; SqCC, squamous cell carcinoma; PCC, papillary cell carcinoma.

Carcinoembryonic antigen-related adhesion molecules (CEAMs) are involved in a number of different processes including cell adhesion, proliferation, differentiation and tumor suppression.^48^ Previous studies have shown that CEAM5 stimulates the progression of NSCLC by promoting cell proliferation and migration and is overexpressed in NSCLC tissues and cells;^49^ and that CEAM6 activates Src-FAK signaling, inhibits anoikis and is overexpressed in LUAD, which correlates with lower overall survival.^50^ In our results, CEAM5 was highly (more than 10 times higher) and CEAM6 was moderately overexpressed in tumor tissue, while we also found changes in the abundance of 3 *N*-glycopeptides. In the sequence of CEACAM5 there is a repeat containing the amino acid sequence of “LQLSNGNR” twice, therefore it cannot be ambiguously determined for this glycopeptide which position the glycan chain is attached to (N197 or N553). For CEAM5 *N*-glycopeptides, the large increase in abundance is likely due to the increase in glycoprotein expression, but for CEAM6 N197 H5N2 the increase in *N*-glycopeptide abundance is much greater than for the glycoprotein. This suggests differential *N*-glycosylation with potential functional consequences, especially as the increase of high-mannose-type glycans have been previously linked to cancers and specifically targeting them in the glycocalyx with lectibodies showed anticancer activity and a potential druggable target.^51^

Haptoglobin (HPT) has been suggested as a serum marker for lung cancer in combination with other proteins,^52^ and HPT *N*-glycoforms, specifically sialylated and fucosylated HPT were also identified as potential lung cancer markers.^53^ In our study, HPT was underexpressed in TWT LUAD, and we also identified decreased levels of H5N4S1 on the N184 *N*-glycosylation site that is reported to carry complex type glycans^54^ in line with our results.

Ras GTPase-activating-like protein IQGAP1 (IQGA1) is a scaffold protein that plays an important role in the assembly and dynamics of the actin cytoskeleton, cell–cell adhesion, and possibly in cell cycle regulation through the regulation of the MAPK pathway. IQGA1 integrates signaling pathways and coordinates several fundamental cellular activities^55^ which results in high oncogenic potential. In lung cancer cells it promotes EGFR-ERK signaling, growth and metastasis,^56^ and its activation can be triggered by the overexpression of the serine/threonine kinase GLK/MAP4K3, which is associated with poor prognosis and recurrence.^57^ We have not detected changes in IQGA1 expression, but we have found that the abundance of the *N*-glycopeptide N1449 F1H5N4S1 decreased substantially in TWT LUAD. Since this glycosylation site was previously unknown, further studies are needed to confirm whether it contributes to the activity of IQGA1.

Keratin, type II cytoskeletal 8 (K2C8) is the major component of the intermediate filament cytoskeleton and is essential for the development and metastasis of various cancers. It has been reported that high K2C8 expression independently predicts poor prognosis for LUAD patients,^58,59^ is a pan-cancer early biomarker,^60^ and that it promotes metastasis and epithelial mesenchymal transition (EMT) via nuclear factor kappa B (NF-κB) signaling.^61^ In our data set, K2C8 was underexpressed in TWT LUAD and we also identified the increased abundance of H5N4S2 on the previously not reported N313 *N*-glycosylation site. Whether this disagreement in K2C8 expression between our data and the literature is specific to TWT LUAD, the differences in experimental techniques (RNaseq and MS-based proteomics), or some other factors needs further investigation.

Laminins are ECM constituents that influence cell differentiation, migration, and adhesion. Laminin subunit alpha-3 (LAMA3) and gamma-2 (LAMC2) are both head and neck cancer specific, and LAMC2 is an unfavorable prognostic marker in lung cancer based on mRNA data. It has also been reported that the upregulation of LAMC2 attenuates the efficacy of anti-PD-1 drugs and is associated with unfavorable outcomes in lung cancer.^62^ We have found both laminin subunits to be under-expressed in TWT LUAD with changes in glycosylation on an unknown site for LAMA3 and a known site of LAMC2 based on UniProt.

Lysosomal alpha-glucosidase (LYAG) is essential in the degradation of glycogen in lysosomes. It has not been extensively studied in the context of lung cancer; however, alpha glucosidase inhibitors have been found to show anticancer activity against NSCLC.^63^ Our results show its increased expression in TWT LUAD which is in line with the increased metabolic activity of tumors,^64^ while the effect of the increased levels of two high mannose glycans on the known N470 site needs further investigation.

Biglycan and decorin (PGS1 and PGS2) are small leucine-rich proteoglycans that have been reported to have pivotal functions in tumor growth and progression through the modulation of receptor-mediated signal transduction.^65^ It has also been shown that the decreased serum level of decorin, which acts as a tumor suppressor, is an independent marker of NSCLC.^66^ We have also found decreased levels of biglycan and decorin in tumor tissue, and we also found that the amount of several PGS1 and PGS2 *N*-glycopeptides decreased by a ratio much higher than that of the expression of the corresponding glycoprotein. This might suggest that changes in *N*-glycosylation on these sites have functional consequences in the context of TWT LUAD.

Transmembrane protein 178B (T178B) a member of the transmembrane protein (TMEM) family have mostly unknown functions; however, experimental evidence suggests that they are tumor suppressors and oncogenes.^67^ There is limited information available specifically on T178B. Here, we report its underexpression in tumor tissue (in line with its tumor suppressor status) and a change in protein glycosylation on a known *N*-glycosylation site, which may further affect protein function.

From a clinical point of view, possibly most important are changes in the abundance of LYAG protein which is a promising therapeutic target as inhibitors for this protein are tested as anticancer agents. However, the inhibitor targets the protein, and little is known about how changes in the glycosylation of this protein might affect therapeutic efficiency or contribute to drug resistance. Additionally, the novel glycosylation site of IQGAP1 protein (N1449) where a decrease in glycopeptide abundance was observed is also interesting as a possible intervention point as this glycoprotein can be linked with sustained proliferative signaling, an important hallmark of cancer.

Limitations of the present study include the relatively low number of samples analyzed and the low tissue amount used for glycopeptide enrichment, resulting in only a few significant changes at the *N*-glycopeptide level. The other reason for the low number of glycopeptide assignments is that only those *N*-glycopeptides were considered that have been confidently and consistently identified in several samples of a given group. Furthermore, these glycopeptides were thoroughly checked and validated using multiple software. Compared to single cell proteomics or glycomics studies, working with FFPE tissue of patient samples still has significant interindividual heterogeneity within tumors, which may be present in the case of our methodology. However, the proteomics analysis identified several proteins that have been previously suggested as promising biomarkers (e.g., PGS2, Cadherin-13) or potential therapeutic targets (e.g., MRE11, MMCM6, SUMO-1) in LUAD indicating that the sample preparation workflow using on-surface digestion and retrospective analysis of tissue samples can yield high-quality and reliable data. These proteins can be considered ideal candidates for drug targeting of this rarely studied type of LUAD in which no known targetable mutations are present. Furthermore, we were able to expand our knowledge with site-specific *N*-glycosylation changes in TWT LUAD and identify novel targets (e.g., IQGA1, T178B). Future studies should therefore focus on analyzing the functional consequences of changes at these identified glycosylation sites, for example, in lung cancer cell lines or 3D cell cultures where specific glycoproteins have been knocked out or the glycosylation machinery has been altered to observe how these affect cell signaling, cell viability, and migrating properties.

## 5 Conclusions

In this study, we conducted a comprehensive proteomics and *N*-glycoproteomics analysis of tumor and adjacent normal tissue in TWT LUAD. Our findings revealed dysregulated biological processes, previously proposed protein biomarkers and several glycoproteins with altered *N*-glycosylation. These results were in line with previously reported protein expression data in most cases; however, we significantly expanded on what is currently known with the novel *N*-glycosylation data. We confirmed and detected changes in glycosylation on previously unconfirmed *N*-glycosylation sites, for example the substantial decrease of the *N*-glycopeptide N1449 F1H5N4S1 of IQGA1, a glycoprotein with high oncogenic potential. As glycosylation influences protein structure greatly, understanding the functional consequences of changes in *N*-glycosylation on important marker or target proteins is of utmost importance. For example, it has been reported that targeting high-mannose-type glycans in the glycocalyx with lectibodies shows anticancer activity, and in our data set, we found the N190 H5N2 peptide of CEAM6, a glycoprotein known to be located in the apical plasma membrane, elevated in tumor tissue.

Whether the reported changes in protein *N*-glycosylation have functional relevance in tumor progression, offer novel points of intervention, or are unique diagnostic approaches needs further investigation. Furthermore, confirming cancer specificity of the changes requires analysis of larger validation cohorts. On the other hand, this is the first glycoproteomics study to focus on TWT LUAD to the best of our knowledge and therefore an important stepping stone toward mapping and understanding its molecular landscape, to eventually offer better solutions to current treatment challenges.

## Supporting Information Available

Annotated tissue slides and reported glycosylation metrics (PDF)
Table S1 listing Byonic proteomics search paramaters, MaxQuant parameters using Byonic_proteomics search results, Byonic parameters for glycopeptide searches, and GlyReSoft search parameters (XLSX)
Table S2 Limma output for all proteins identified using MaxQuant (XLSX)
Table S3 Results of the GSEA using ClusterProfiler (XLSX)
Table S4 Complete list of *N*-glycopeptides quantified using GlycReSoft (XLSX)
