# Supplementary material for: Glycoproteomics Analysis of Triple Wild-Type Lung Adenocarcinoma Tissue Samples
Source: J Proteome Res. 2025 Apr 2;24(5):2419–29. doi: 10.1021/acs.jproteome.4c01063 (PMC12053933; doi:10.1021/acs.jproteome.4c01063)
Supplement: Supplementary file 1 — pr4c01063_si_001.pdf [file pr4c01063_si_001.pdf]

Glycoproteomics analysis of triple wild-type lung adenocarcinoma tissue samples

Simon Nándor Sugár[1]*, Balázs András Molnár[1], Fanni Bugyi[1,2], Gábor Kecskeméti[3], Zoltán Szabó[3], Ibolya Laczó[4], Tünde Harkó[5], Judit Moldvay[5,6], Lilla Turiák[1]*

[1]MTA-TTK Lendület (Momentum) Glycan Biomarker Research Group, HUN-REN Research Centre for Natural Sciences, Magyar Tudósok krt. 2., Budapest, H-1117, Hungary

[2]Hevesy György PhD School of Chemistry, ELTE Eötvös Loránd University, Pázmány Péter sétány 1/A, Budapest, H-1117, Hungary

[3]Department of Medical Chemistry, Albert Szent-Györgyi Medical School, University of Szeged, Dóm square 8, Szeged, H-6720, Hungary

[4]Békés County Central Hospital, Semmelweis u.1, Gyula, H-5700, Hungary

[5]National Korányi Institute of Pulmonology, Korányi Frigyes str. 1, Budapest, H-1121, Hungary

[6]Pulmonology Clinic, Albert Szent-Györgyi Medical School, University of Szeged, Alkotmány str. 36., Deszk, H-6771, Hungary

Supplementary material

Supplementary Figure S1

Annotated tissue slides with tumour sections (marked red) and adjacent normal sections (marked green).

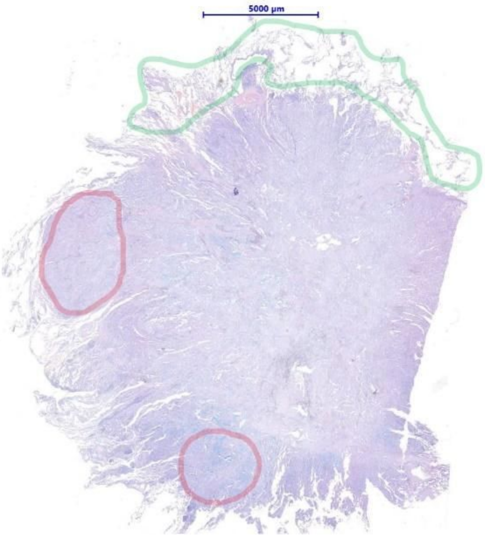

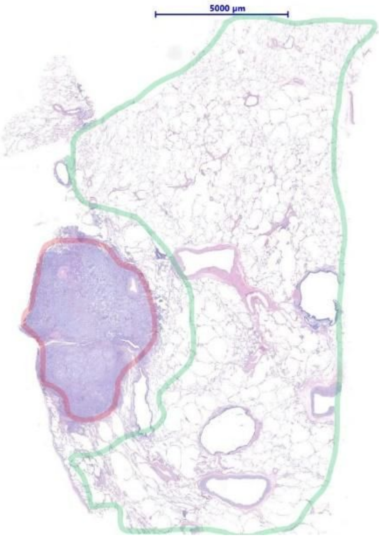

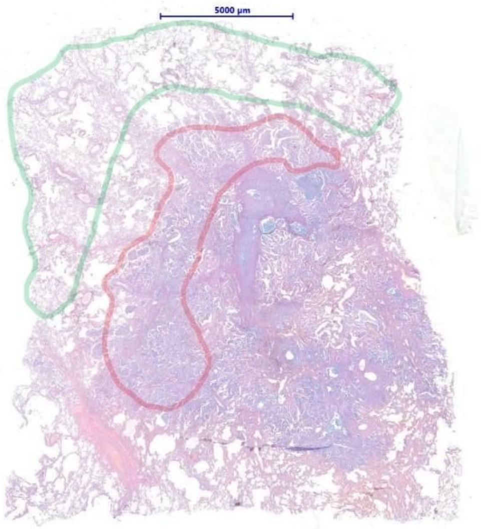
5000 µm

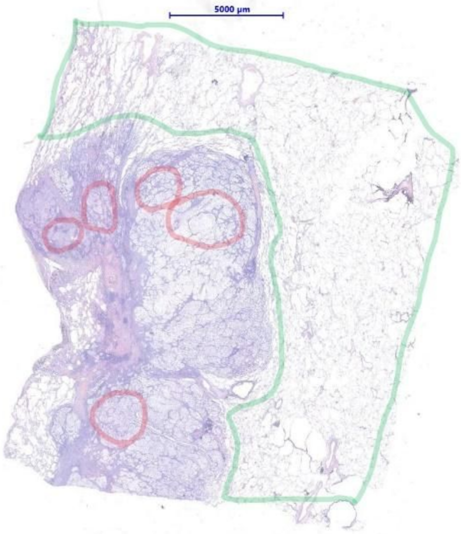
5000 µm

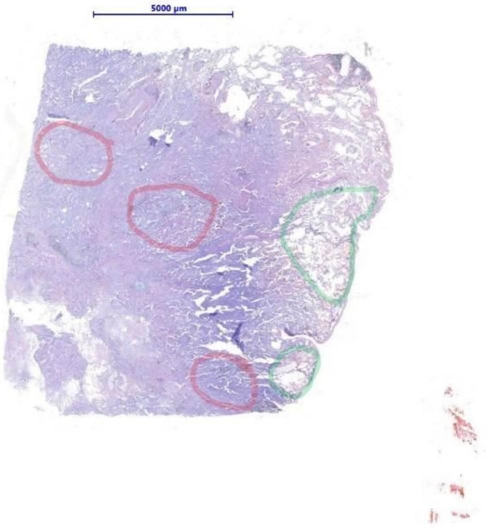
5000 µm

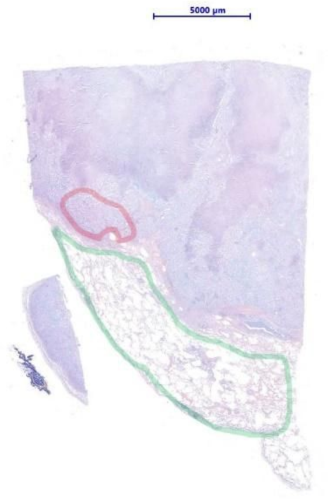
5000 µm

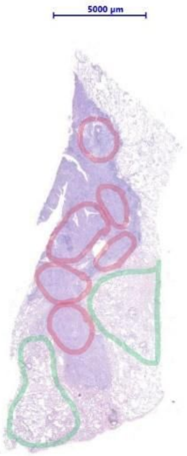
5000 µm

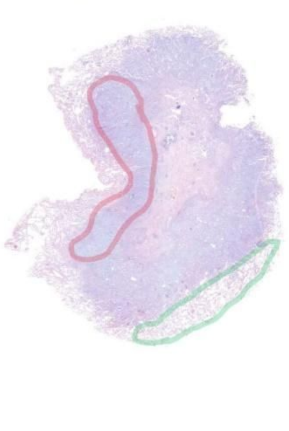
10000 µm

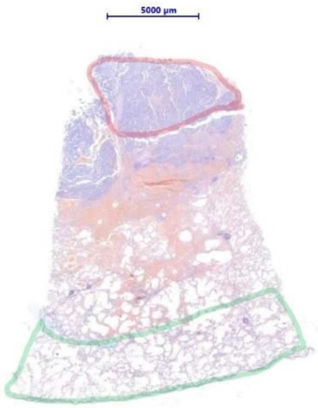
5000 µm

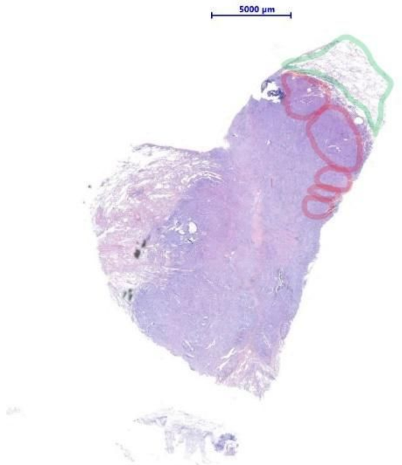
5000 µm

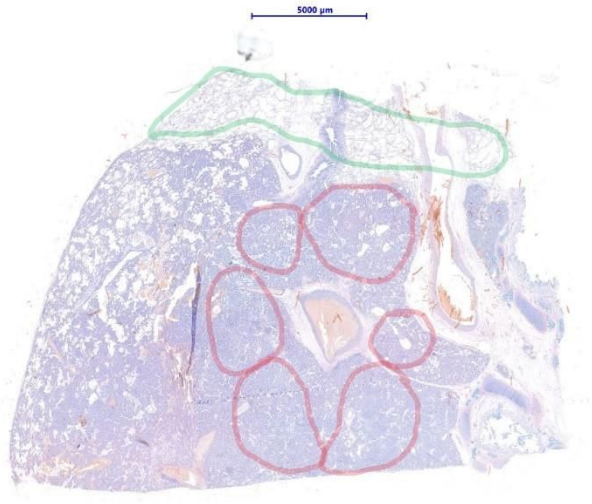
5000 µm

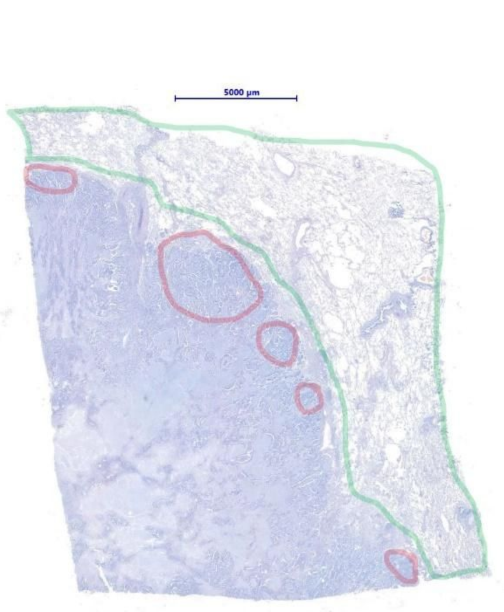
5000 µm

Supplementary equations 1-6

Glycosylation metrics reported in the original manuscript. M_{Sia}, M_{Gal}, M_{Fuc} represents *Sialylation, Galactosylation, Fucosylation* respectively, while $M_{SiaTotal}$, $M_{GalTotal}$, $M_{FucTotal}$ represents the overall metrics.

$$M_{Sia} = Intensity \times \frac{Antennae_{Sia}}{Antennae_{Total}} \qquad \text{Equation 1}$$

$$M_{Gal} = Intensity \times \frac{Antennae_{Gal}}{Antennae_{Total}} \qquad \text{Equation 2}$$

$$M_{Fuc} = Intensity \times \frac{Number\ of_{Fuc}}{Max\ number\ of_{Fuc}} \qquad \text{Equation 3}$$

$$M_{SiaTotal} = \frac{\sum Intensity \times \frac{Antennae_{Sia}}{Antennae_{Total}}}{\sum Intensity} \qquad \text{Equation 4}$$

$$M_{GalTotal} = \frac{\sum Intensity \times \frac{Antennae_{Gal}}{Antennae_{Total}}}{\sum Intensity} \qquad \text{Equation 5}$$

$$M_{FucTotal} = \frac{\sum Intensity \times \frac{Number\ of_{Fuc}}{Max\ number\ of_{Fuc}}}{\sum Intensity} \qquad \text{Equation6}$$
